# Characteristics of South African patients presenting with kidney disease in rural KwaZulu-Natal: a cross sectional study

**DOI:** 10.1186/1471-2369-15-61

**Published:** 2014-04-14

**Authors:** Nomandla D Madala, Gertrude P Thusi, Alain G H Assounga, Saraladevi Naicker

**Affiliations:** 1 Department of Nephrology, Division of Internal Medicine, Nelson R Mandela School of Medicine, University of KwaZulu-Natal, Durban, South Africa; 2 Department of General Internal Medicine, Division of Internal Medicine, Nelson R Mandela School of Medicine, University of KwaZulu-Natal, Durban, South Africa; 3 Division of Nephrology, Department of Internal Medicine, Faculty of Health Sciences, University of the Witwatersrand, Johannesburg, South Africa

**Keywords:** Chronic kidney disease, Diabetes, Dyslipidaemia, HIV, Hypertension, Rural, South Africans

## Abstract

**Background:**

Diabetes mellitus is the leading cause of end-stage renal disease (ESRD) globally. Diabetes and human immunodeficiency virus (HIV), both prevalent in South Africa, have not been reported as significant causes of ESRD.

**Methods:**

We evaluated chronic kidney disease (CKD) and cardiovascular disease risk factors in a cross-sectional study of 302 patients (165 females/ 137 males) at a CKD clinic in rural northern KwaZulu-Natal. We included all CKD outpatient clinic attendees and excluded acute renal failure patients. Demographic, clinical and laboratory data collected were analyzed with Stata11 software. Logistic regression analysis was used to determine factors associated with advanced CKD and results expressed as the odds ratio with the 95% confidence interval [OR (95% CI)].

**Results:**

Of 302 patients analyzed, 290 (96%) were black African. Mean age ± SD was 47.1 ± 17.0 years. Approximately 86.4% of females and 54.5% of males were overweight/ obese. Dyslipidaemia was observed in 47.9% females and 29.2% males (P < 0.001). Estimated glomerular filtration rate (eGFR) was <30 ml/min/1.73 m^2^ in 50.6% patients. CKD risk factors observed were: hypertension (77.8%), diabetes (29.8%), HIV (28.5%), glomerulonephritis (7.0%) and tubulointerstitial diseases (5.6%). Independent factors associated with eGFR <30 ml/min/1.73 m^2^ at presentation were: HIV [OR = 2.4 (1.3-4.2), P = 0.004] and hypertension [OR = 2.3 (1.3-4.2), P = 0.007].

**Conclusion:**

Diabetes and HIV are prevalent in CKD patients at primary/regional level healthcare in South Africa. With registry data lacking, dedicated CKD clinics at lower healthcare levels may provide valuable data on CKD epidemiology including changes in aetiology. Primary healthcare practitioners are faced with advanced CKD patients in resource-poor settings, with limited opportunity for upward referral hence the need for nephrology outreach programs.

## Background

Hypertension and glomerulonephritis were the major causes of end-stage renal disease (ESRD) among South Africans in previous registry data [1]. In contrast, data have shown type 2 diabetes as the commonest cause of ESRD globally, accounting for up to 40% [2]. This has been in parallel with the global increase in the prevalence of obesity and type 2 diabetes. The prevalence of obesity and diabetes in South Africa has been reported to be high [3]. Data have also shown that mortality from diabetes increased by 38% in the period from 1999 to 2006 with an even greater increase of 67% reported for mortality due to kidney diseases [4]. Diabetic patients have been under-represented in registry data hence accurate data on the prevalence of diabetes in the South African ESRD population are lacking. A study in the Western Cape province reported that <20% of diabetic patients assessed for renal replacement therapy (RRT) between 1988 and 2003 were offered RRT, consequently diabetic patients only comprised 6.2% of accepted patients overall [5].

Human immunodeficiency virus (HIV) infection is another ESRD cause under-represented in local data since HIV patients were previously excluded from RRT and are only recently being offered RRT. [6] The socioeconomic and health consequences of CKD are well-documented globally [7,8]. Apart from the high costs of RRT, the pressure on national resources is further compounded by the high cardiovascular disease (CVD) burden observed in CKD patients. Consequently, concerted global efforts aimed at CKD screening and early diagnosis have been called for [9]. Screening of the general population for CKD has not been considered cost-effective hence many CKD screening programs often target individuals and groups characterized by a high CKD prevalence [10]. Variable success rates have been reported from countries worldwide, including South Africa, with the best prospects for sustainability observed in programs that could be incorporated into national health policy [11,12]. Therefore, epidemiological data are needed in South Africa to provide the necessary framework for incorporating CKD early detection and management into primary level healthcare with other chronic non-communicable diseases. Our aim was to describe the prevalence of CKD and CVD risk factors and determine factors associated with CKD severity in patients presenting at a CKD clinic in the predominantly rural northern KwaZulu-Natal region, South Africa.

## Methods

### Study design and setting

This was a cross-sectional analysis of records kept at the clinic of consecutive outpatients seen at the dedicated CKD clinic at Ngwelezana hospital situated in the Uthungulu district, 5 km from the town of Empangeni and approximately 200 km north of Durban, in KwaZulu-Natal province, South Africa. The hospital provides district (primary) level and regional (secondary) level healthcare services to 20 district hospitals that provide primary healthcare services to the approximately 2 million people living in the northern KwaZulu-Natal districts of Uthungulu, Zululand as well as Umkhanyakude. The majority live in rural areas with the proportion of the population living in urban areas estimated at 14.5%, 13.4% and 3.8%, for Uthungulu, Zululand and Umkhanyakude, respectively [13]. The region is largely poorly-resourced with the latter 2 ranked among the 10 most deprived districts in South Africa in 2007 [14]. Only acute peritoneal dialysis through a rigid catheter was offered at Ngwelezana hospital and only a minority of patients could access RRT in the academic tertiary level nephrology unit in Durban. The clinic, established by a specialist physician in 2008 to improve early CKD identification, had junior doctors, nurses, a social worker, dietician and visiting nephrologist weekly initially, with less frequent visits later. The University of KwaZulu-Natal Biomedical Research Ethics Committee approved the study.

### Patients

We reviewed records, kept at the CKD clinic, of all patients seen from 31^st^ January 2008 to 31^st^ January 2011. All patients presenting to the clinic were referred by primary healthcare doctors from any of the 20 district hospitals in the geographic area served by the hospital. Referral was at the discretion of the primary healthcare doctor and each patient presented with a referral letter from the referring doctor providing details of the clinical diagnosis made, results of investigations performed at the referring hospital as well as their current medication. All referrals to our clinic were for kidney disease based on clinical as well as structural evidence of kidney disease noted at the referring hospital, including proteinuria and elevated serum creatinine levels. At first presentation to our clinic, all patients underwent clinical assessment (history and examination) and laboratory investigations were done as part of standard care. Renal disease aetiology was largely determined clinically as renal histology was unavailable except where glomerulonephritis unrelated to HIV was suspected and those patients were referred to the tertiary center in Durban for a renal biopsy. Renal ultrasound services were available intermittently and were accessed when that was feasible.

*Inclusion criteria were*: Patients with a diagnosis of CKD

*Exclusion criteria were*: Diagnosis of acute renal failure

### Data collection

The following data were recorded for each patient.

a. *Demographic characteristics*: Age, gender, ethnicity, source of referral, area of residence and smoking status, where ethnicity was used according to Census population classification data: African (black), white, Indian and coloured [13]. Referral source was categorized according to location of the referring hospital into: (i) Uthungulu district, in which the CKD clinic was located, (ii) Umkhanyakude and (iii) Zululand. Patients were classified as urban or rural based on Census definitions of their place of residence [13]. Smoking was recorded as (i) never smoked/ex-smoker and (ii) current smoker.

b. *History*: Medical history, such as hypertension, diabetes, HIV, dyslipidaemia and medication. Patient records, referral documents, current medication and pharmacy entries were used in conjunction with self-reported history to establish the diagnosis.

c. *Physical examination*: Anthropometric measures [weight (kg), height (cm), waist circumference (cm) and body mass index = weight/height^2^ (kg/m^2^)], blood pressure (mmHg) and dipstick analysis for proteinuria. Overweight/obese was defined as presence of BMI ≥25 kg/m^2^ and/or waist circumference ≥80 cm in females and ≥92 cm in males.

d. *Laboratory tests*: Results of blood tests recorded were - serum creatinine, total cholesterol, serum albumin and haemoglobin. Total cholesterol >5.0 mmol/l was included in dyslipidaemia definition. Serum creatinine results were obtained prior to the implementation of the IDMS-traceable assay and values were not recalibrated to be IDMS-traceable. The abbreviated Modification of Diet in Renal Disease (MDRD) and Schwartz equations were used to calculate eGFR in patients aged ≥18 years and <18 years, respectively:

1. MDRD-eGFR (mL/min/1.73 m^2^) = 186 × (Serum creatinine/88.4)^-1.154^ × (Age)^-0.203^ × (0.742 if female) × (1.212 if African American) [15]

2. Schwartz ‒ eGFR (mL/min/1.73 m^2^) = k x (height in cm) ÷ Serum creatinine [16]

The African-American coefficient was omitted following evidence that this improved MDRD-eGFR equation accuracy in Africans [17-19].

#### Definitions

CKD was defined by eGFR <60 ml/min/1.73 m^2^ and/or proteinuria and/or abnormal renal ultrasound, persistent for ≥3 months.

Acute renal failure was defined by complete recovery of kidney function on subsequent visits after initial presentation.

### Statistical analysis

Intercooled Stata version 11 (Texas, USA) was used for data analysis. Categorical data were described as proportions and males compared with females using the chi-square test. Continuous data were summarized as mean ± standard deviation (SD) with the t-test used to assess differences between males and females. Non-normal data were expressed as median (interquartile range) and differences between the two groups were evaluated using the Mann–Whitney test. Odds ratios (95% CI) were calculated using logistic regression analysis to evaluate the factors associated with presenting with a low eGFR (<60- and <30 ml/min/1.73 m^2^).

## Results

A total of 313 CKD patients, 174 (55.6%) females and 139 (44.4%) males, were seen on their first visit during the study period (mean age 47.1 ± 17.0 years). Data were available for 302/313 (96.5%) patients that were included in the analysis and 11 patients were excluded due to unavailability of clinical and/or laboratory data. The ethnic distribution of study patients was: 290/302 (96%) African (black), 4 (1.3%) Indian and 8 (2.7%) white. This closely resembled the population distribution in the 3 districts comprising northern KwaZulu-Natal [13]. Almost two-thirds, 191 (63.3%) were referred from Uthungulu district while 85 (28.2%) and 26 (8.6%) were from various hospitals across Umkhanyakude and Zululand districts, respectively. Over half, 165 (54.6%) were classified as urban and 137 (45.4%) as rural. Table 1 shows clinical and laboratory characteristics at presentation by gender. Significantly more males than females reported current smoking while females were more likely than males to have the CVD risk markers of being overweight/obese and having dyslipidaemia. Over 70% presented with stage 3 CKD or worse with the presenting median eGFR (IQR) being 28.9 (49.8) ml/min/1.73 m^2^.

**Table 1:** Distribution of demographic, clinical and laboratory data of study patients by gender

**Parameter**	**Male *n = 137***	**Female *n = 165***	**P-value**
**African**	130 (94.9%)	160 (97.0%)	0.282
**Rural**	60 (43.8%)	77 (46.7%)	0.618
**Smoking**^**#**^	24/134 (17.9%)	2/158 (1.4%)	<0.001
**Age (years)**	45.0 ± 17.6	48.5 ± 16.8	0.055
**Systolic blood pressure (mmHg)**	144.6 ± 28.3	141.1 ± 25.5	0.362
**Diastolic blood pressure (mmHg)**	84.2 ± 18.1	81.0 ± 19.0	0.153
**BMI (kg/m**^**2**^**)**^**#**^	25.4 ± 5.5	29.4 ± 7.9	<0.001
**Waist circumference (cm)**^**#**^	92.5 ± 15.2	98.5 ± 20.6	0.021
**Overweight/obese**^**#**^	55/101 (54.5%)	108/125 (86.4%)	<0.001
**(*% with data*)**	(*73.7*)	(*75.8*)	
**Dyslipidaemia**	40 (29.2%)	78 (47.9%)	0.001
Total cholesterol (mmol/l)*	4.1 (3.5-5.1)	4.5 (3.7-6.0)	0.009
Serum creatinine (μmol/l)*	215 (116–464)	173 (90–336)	0.009
**MDRD-eGFR (ml/min/1.73 m**^**2**^)*	28.5 (11.8-61.9)	30.4 (13.7-63.5)	0.906
**% with MDRD-eGFR <60**	100 (73.0%)	119 (72.6%)	0.933
**% with MDRD-eGFR <30**	68 (49.6%)	85 (51.5%)	0.745
**% with MDRD-eGFR <15**	44 (31.9%)	49 (29.9%)	0.707
**Proteinuria**	66 (48.2%)	68 (41.2%)	0.225
**Serum albumin (g/l)**	30.6 ± 9.6	31.3 ± 8.8	0.530
**Haemoglobin (g/dl)**	10.7 ± 2.7	10.3 ± 2.4	0.183

^#^Variables with incomplete data; the proportions with available data for smoking, BMI and waist circumference were 97.8%, 54% and 66.4%, respectively, for males while for females data were available in 95.8%, 59.4% and 67.9%, respectively.

Continuous data were summarized as mean ± SD and non-normal data* as median (IQR); the 25^th^-75^th^ percentiles are specified rather than the range. Categorical data were expressed as number (%).

### Aetiology of CKD and co-morbid risk factors

**Figure 1:**
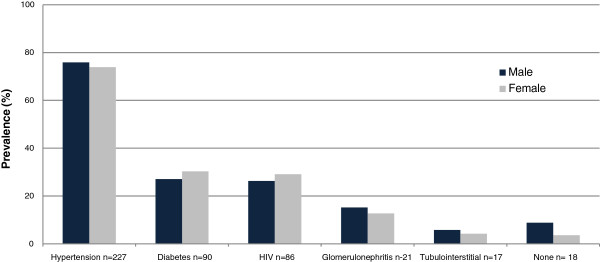
Prevalence of CKD risk factors in the 302 study patients.

**Figure 2:**
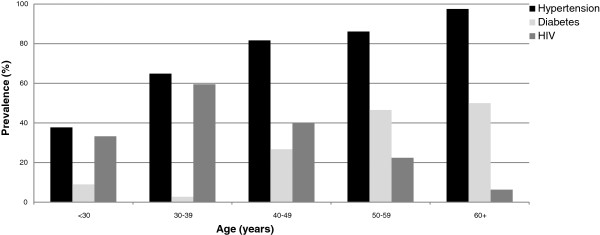
Distribution of hypertension, diabetes and HIV by age group.

**Figure 3:**
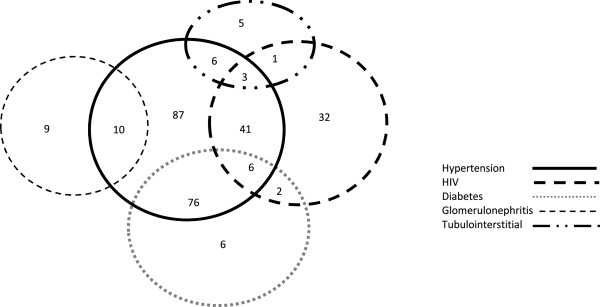
Distribution and overlap of major CKD risk factors in patients with one or more co-morbidities.

Figure 1 shows that the 3 commonest diagnoses encountered were: hypertension in 227 (75.2%), diabetes in 90 (29.8%) and HIV in 86 (28.5%) with similar gender distribution. Self-reported history was verified in all patients using medical records. Renal diagnoses in 21 patients referred for renal biopsy were: primary glomerulonephritis (16), hepatitis B virus-associated (4) and lupus nephritis (1). Tubulointerstitial diseases were observed in 17 (5.6%) with autosomal dominant polycystic kidney disease (11), obstructive uropathy (5) and presumed analgesic nephropathy (1). Hypertension and diabetes increased with age while HIV was prevalent in young patients (Figure 2). Multiple concomitant CKD risk factors were common (51.1%); dual co-morbidities in 136 (47.9%) patients and triple co-morbidities in 9 (3.2%). CKD was attributed to a single cause in 139 (48.9%) patients; 87/302 (28.8%) had hypertension alone, 32/302 (10.6%) had HIV alone while glomerulonephritis alone and tubulointerstitial disease were uncommon; 9/302 (3.0%) and 5/302 (1.7%), respectively (Figure 3). Only 6/90 (6.7%) diabetic patients had diabetes alone with concurrent hypertension in 76 (84.4%) and concurrent HIV in 8 (8.9%). Hypertension was a co-morbid diagnosis in >50% of patients with HIV, glomerulonephritis and tubulointerstitial disease as well (Figure 3).

### Other cardiovascular disease risk factors

Dyslipidaemia was found in 118 (39.1%) patients overall and there was no difference in the various CKD stages (P = 0.637). In 226 patients with available data to determine overweight/obese status, prevalence of dyslipidaemia was 44.2% in overweight/obese patients and 28.6% in those who were not, P = 0.032. Fifteen patients (6 males and 9 females) had CVD complications; stroke (8), congestive cardiac failure (6) and atherosclerotic renovascular disease (1).

### Factors associated with presenting with advanced CKD

**Figure 4:**
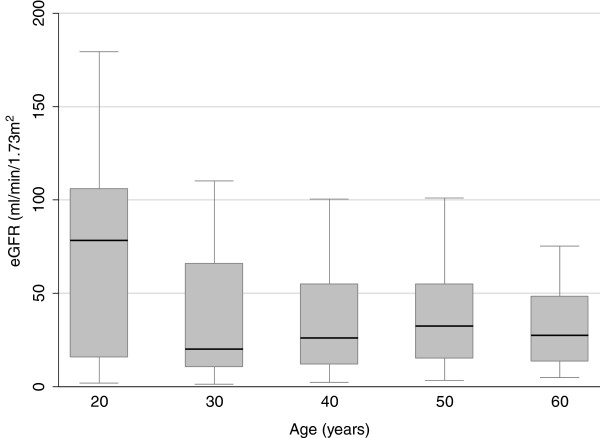
**Distribution of patients grouped according to presenting eGFR level and age.** The box-and-whisker plot shows the 25^th^, 50^th^ and 75^th^ percentiles in various age groups.

Figure 4 shows that eGFR was highest in younger patients and decreased with increasing age [OR = 0.48 (0.37-0.62) per 10-year, P <0.001] but there was no significant age difference in those presenting with eGFR <15 ml/min/1.73 m^2^ (P = 0.099). Using logistic regression analysis, independent factors associated with eGFR <60 ml/min/1.73 m^2^ at presentation were: age ≥60 years [OR = 4.5 (2.0-10.5)], HIV infection [OR = 3.0 (1.5-5.9], hypertension [OR = 2.7 (1.4-5.0] and rural residence [OR = 1.9 (1.1-3.4)] while a lower presenting eGFR <30 ml/min/1.73 m^2^ was strongly associated with HIV as well as hypertension but not with age or residence (Table 2). Disease severity at presentation was not significantly associated with gender, diabetes or referral source.

**Table 2:** **Multivariable logistic regression analysis results showing factors associated with presenting eGFR <60 and <30 ml/min/1.73 m**^**2**^

**Risk factor**	**MDRD–eGFR <60**		**MDRD–eGFR <30**	
	**Adjusted OR (95% CI)**	**P–value**	**Adjusted OR (95% CI)**	**P–value**
**Age ≥60 years**	4.5 (2.0–10.5)	<0.001	1.4 (0.8–2.5)	0.261
**HIV**	3.0 (1.5–5.9)	0.001	2.4 (1.3–4.2)	0.004
**Hypertension**	2.7 (1.4–5.0)	0.002	2.3 (1.3–4.2)	0.007
**Rural**	1.9 (1.1–3.4)	0.021	1.5 (0.9–2.4)	0.100
**Diabetes**	1.7 (0.8–3.3)	0.148	1.3 (0.7–2.2)	0.415
**Male gender**	1.2 (0.7–2.1)	0.493	1.0 (0.6–1.6)	0.998
**Referral source**	1.3 (0.9–1.8)	0.131	1.2 (0.9–1.5)	0.274

## Discussion

This is the first study in South Africa to report high prevalence estimates for diabetes and HIV, 29.8% and 28.5%, respectively in a single patient cohort at a secondary level healthcare-based dedicated CKD clinic. Our observations add important data on CKD epidemiology outside of a tertiary level nephrology environment, particularly because diabetes and HIV have mostly been under-represented in previous studies. In two recent studies, diabetes was observed in 10.8% of patients in the Free State province and <5% of renal biopsies in the Western Cape province while HIV-associated nephropathy (HIVAN) was high in the latter study, comprising 25.7% of annual renal biopsies in 2009 [20,21]. Most HIV patients in the present study were presumed to have HIVAN since renal biopsy was largely not accessible, which was based on a previous study from our institution that showed HIVAN in >80% of HIV patients with microalbuminuria or overt proteinuria [22].

Dedicated CKD clinics typically conducted at tertiary level by nephrologists were inaccessible to most patients in this study due to various healthcare resource constraints hence the establishment of this CKD outreach clinic. Published South African experience of nephrology outreach is limited to a study in Soweto primary healthcare clinics in Gauteng province that found eGFR <60 ml/min/1.73 m^2^ in 26% and nephrotic-range proteinuria in 9% of patients evaluated [12]. The high proportion of patients with advanced disease (50.6% with eGFR <30 ml/min/1.73 m^2^) in our clinic highlights an important reality in resource-poor settings that CKD patients remain at district and regional level healthcare unable to access tertiary care, thus primary healthcare practitioners are managing increasingly complex patients.

The strong association of HIV and hypertension with a low presenting eGFR level in this study may be due to late referral or it may suggest a high risk for CKD progression in this population. In a recent systematic review, independent risk factors for late referral in CKD included older age, multiple comorbidities, renal disease aetiology and lower socioeconomic status [23]. Older age and rural residence (an indicator of poor socioeconomic status) were independent factors associated with presenting with stage 3 CKD or worse in our study. Only 45% of clinic patients were classified as rural while over 85% of the 2 million population served by our hospital were rural. The discrepancy may be because >60% of referrals were from within the district where the CKD clinic is located, which might suggest disproportionately greater access to the clinic by the urban minority population or a higher CKD prevalence in urban communities; both postulates need further evaluation in population-based studies. The strong association observed between older age and lower eGFR remained significant even after adjusting for hypertension and diabetes, both of which increased in prevalence with age. Hypertension prevalence also increases with worsening CKD, which was also observed in the present study with hypertension found in 80.5% in patients with stage 3 CKD and 85% of those with stage 5 CKD. These findings were in keeping with observations in the Kidney Early Evaluation Program in Americans where hypertension prevalence was 86.8% in stage 3 CKD, increasing to 95.5% in stages 4–5 [24].

The high prevalence of advanced CKD in the our study could reflect rapid CKD progression as patients were almost exclusively black (96%) thus at high risk for more severe hypertensive target organ damage, including CKD, as reported in black South Africans [25]. However, CKD progression could not be evaluated in this present study design. Hypertension alone was again shown in this study as the commonest cause of CKD, as in many other studies in black South Africans and other sub-Saharan populations [20,26-29]. Although clinical diagnosis was adopted in this study, there is evidence from published biopsy data for hypertensive nephrosclerosis as the major ESRD cause in black South Africans [26]. Studies in African-Americans showed that African ancestry (black race) is also associated with aggressive CKD in HIV patients. HIV infection in black patients was associated with a greater risk for incident CKD, higher prevalence for more aggressive disease with faster progression to ESRD and worse CKD outcome than in white patients [30]. Studies are needed in South Africans to evaluate progression of CKD in HIV patients, particularly as CKD patients with HIV were younger (mean age 39.5 ± 11.9 years in HIV patients versus 47.1 ± 17.0 years in the overall cohort). The weak association of diabetes with CKD observed in the present study contrasts with the overwhelming evidence in the literature and was probably because too few patients (<10%) had diabetes alone. This could reflect under-recognition or lack of referral of patients with diabetic CKD without concurrent hypertension but there are no published studies in this region to support this speculation thus far. Elsewhere, under-recognition of diabetic CKD has been identified as a barrier yet to be overcome in the global efforts against CKD, with some surveys in the United States putting patient awareness of their disease as low as 9.4% in those with diabetic CKD [2]. Patients with suspected glomerulonephritis (non-HIV) in our province tend to be referred directly to the tertiary centre hence the low prevalence in this study differs from previous reports of glomerulonephritis as a common cause of CKD in sub-Saharan Africa [1,5,27,29,31].

The proportion of patients that were overweight/ obese in this study (86.4% in females and 54.5% in males) was substantially higher than the reported prevalence of 58.5% and 25.4% in black South African women and men, respectively, in the general population [3]. Our observations were similar to findings in the Soweto study cited earlier, which reported that 86% of females and 40.9% of males were overweight/obese using waist circumference while prevalence was 83% and 75%, respectively, when using a composite measure of waist circumference combined with BMI [12]. Our study is the first to report a high prevalence of dyslipidaemia (39.1%) in South African CKD patients. The results are in keeping with the prevalence of dyslipidaemia or hypercholesterolaemia of 38.4% in sub-Saharan Africa reported in a recent meta-analysis of 16 studies of high cardiovascular-risk patients [32]. However, using total cholesterol to evaluate dyslipidaemia, as in our clinic, may underestimate the true burden since HDL, triglycerides and apolipoproteins are the major abnormalities found in CKD [33].

### Study strengths and limitations

This study provides important insight into CKD and CVD risk factors in this population that has not been studied before. The results add to the much needed CKD epidemiology data in South Africa in the absence of RRT registry data and representative population studies. The study is subject to the inherent limitation of a cross sectional design in which causal associations cannot be determined between CKD severity and the various risk factors as the temporal sequence is unknown. Selection bias may have potentially occurred from preferential referral of advanced CKD patients over those with earlier CKD and contributed to the small sample size thus results may not reflect the true prevalence in northern KwaZulu-Natal. Another limitation is the use of serum creatinine values without re-calibration to the IDMS assay as this could have introduced bias in calculating eGFR. Correction to the IDMS method substantially improves accuracy of MDRD-eGFR, especially in CKD stages 1–3 although the impact of correction was shown to be minimal at GFR <45 ml/min/1.73m^2^[34]. We expect the bias from non-standardized creatinine to be small in this study, as the median eGFR was low. This will be eliminated in future studies as all laboratories nationally now use standardized serum creatinine in calculating eGFR. The validity of MDRD eGFR in HIV patients is uncertain thus using MDRD eGFR in our HIV patients may be a potential limitation. There are no validation studies in our population and data from published validation studies in various populations are inconclusive on the equation that provides the best GFR estimate. A recent systematic review of studies that evaluated MDRD eGFR reported that the performance was similar in HIV-infected and HIV-negative patients [35]. The absence of renal histology, CD4 cell count and antiretroviral (ARV) therapy data may also be a limitation since these may be significant risk factors for incident CKD as well as progression.

### Study implications

The implications of our findings of advanced CKD in the majority (>70%) of patients, in strong association with HIV infection and hypertension, is that patients presenting with these conditions should be screened for CKD at initial presentation to primary healthcare practitioners and regularly thereafter. The high HIV burden in South Africa as well as the widespread national ARV therapy roll-out program with the resultant improving patient survival means that HIV is likely to become a significant contributor to the CKD burden. Further research is needed to determine the public health impact including resource requirements for CKD early detection and management. The successful integration of HIV care into the primary healthcare system nationally, even in resource-poor settings, provides a promising model for CKD as well, hence integration of CKD screening and management into the existing HIV infrastructure needs to be explored as a potentially feasible strategy in managing CKD.

## Conclusion

Our results provide evidence for the first time in South Africa that diabetes and HIV are prevalent in CKD patients at primary/regional level suggesting that both may be emerging as significant causes of CKD in South Africans, following hypertension. The high prevalence of advanced CKD and other CVD risk factors in this cohort suggests that increasingly complex patients are being managed at lower healthcare levels in this region, a trend we expect to be more widespread in the country because nephrology services are available only in a few cities. This underscores the need for dedicated CKD clinics with specialist outreach support in resource-poor areas where options for upwards referral and RRT are limited.

## Competing interests

The authors declare that they have no competing interests.

## Authors’ contributions

NDM conceived the study while working as the visiting nephrologist, participated in writing the protocol, data collection and interpretation as well as in writing the draft manuscript. GPT conceptualized and established the clinic in 2008 while working as a specialist physician, participated in data collection and in writing the draft manuscript. AGHA provided training for GPT during establishment of the clinic and provided intellectual input in writing the study protocol as well as the manuscript. SN provided critical intellectual input into the study protocol and in revising the draft manuscript. All authors read and approved the final manuscript, except GPT.

## Pre-publication history

The pre-publication history for this paper can be accessed here:

http://www.biomedcentral.com/1471-2369/15/61/prepub
